# LncRNA RP11-620J15.3 promotes HCC cell proliferation and metastasis by targeting miR-326/GPI to enhance glycolysis

**DOI:** 10.1186/s13062-023-00370-0

**Published:** 2023-04-05

**Authors:** Chuanjiang Liu, Kequan Xu, Jiayin Liu, Chao He, Pan Liu, Qiang Fu, Hongwei Zhang, Tao Qin

**Affiliations:** 1 Department of Hepatobiliary and Pancreatic Surgery, Zhengzhou University People’s Hospital (Henan Provincial People’s Hospital), Zhengzhou, 450003 People’s Republic of China; 2 Department of Hepatobiliary and Pancreatic Surgery, Zhongnan Hospital of Wuhan University, Wuhan, 430071 People’s Republic of China

## Abstract

**Background:**

Accumulating studies have demonstrated that the Warburg effect plays a central role in the occurrence and development of hepatocellular carcinoma (HCC), albeit the role of non-coding RNA (lncRNA) in its association remains unclear.

**Methods:**

The Zhengzhou University People’s Hospital kindly provided 80 pairs of HCC tissues and their matched paracancerous tissues for this study. Bioinformatics analysis, real-time quantitative polymerase chain reaction, Western blotting, and oncology functional assays were performed to determine the contribution of RP11-620J15.3 to the development of HCC. The mechanism of co-immunoprecipitation and a luciferase reporter gene was employed to ascertain how RP11-620J15.3 interacts with important molecular targets.

**Results:**

Our results revealed that a lncRNA termed RP11-620J15.3 was overexpressed in HCC and was substantially associated with the tumor size. A high expression of RP11-620J15.3 mRNA was found to be significantly associated with worsening prognosis in HCC patients. We discovered that RP11-620J15.3 stimulated the glycolytic pathway in HCC cells by RNA-sequencing (RNA-seq) and metabolomics analyses. Mechanistically, RP11-620J15.3 acted as a competitive endogenous RNA to regulate the GPI expression by sponging miR-326 in HCC. In addition, TBP acted as a transcription factor for RP11-620J15.3, which contributed to the high expression of RP11-620J15.3 in HCC cells.

**Conclusion:**

Based on our findings, lncRNA RP11-620J15.3 is a novel LncRNA that positively regulates tumor progression. Specifically, RP11-620J15.3/miR-326/GPI pathway promotes HCC malignant progression by regulating glycolysis, thereby providing novel targets for HCC treatment and drug development.

**Supplementary Information:**

The online version contains supplementary material available at 10.1186/s13062-023-00370-0.

## Introduction

Globally, hepatocellular carcinoma (HCC) ranks fifth in cancer incidence and third in cancer-related mortality [1, 2]. Although surgery, radiofrequency ablation, transarterial chemoembolization, targeted therapy, and emerging immunotherapies have made great therapeutic strides in recent years, the survival rate of HCC patients can be further improved [3–6]. Thus, a new theoretical system of biological markers is urgently needed for use in a clinical setting to improve the early diagnosis and subsequent development of targeted therapy for HCC as well as to improve the survival rate of HCC patients.

According to recent research, metabolic reprogramming is crucial to cancer progression. Particularly, the Warburg effect, wherein cancer cells are dependent on glucose as a substrate under aerobic conditions, is important to the pathogenesis of HCC [7–10]. The Warburg effect regulates HCC proliferation, immune escape, invasion, metastasis, angiogenesis, and drug resistance. In terms of the development of cancer cells, the Warburg effect enhances glycolysis and glutaminolysis to sustain cell proliferation, which subsequently increases biomass and creates an acidic microenvironment that is promising for cell migration and invasion [11–13]. Over time, this pathological metabolism induces epigenetic and genetic modifications, which further prompt cancer cell growth and aggressiveness. The Warburg effect activation has thus been considered the driving force of HCC progression, albeit its specific underlying molecular mechanism remains unclear.

Long-noncoding RNAs (lncRNAs) are a class of RNAs of length > 200 bases, and their transcripts are evolutionarily conserved [14, 15]. These transcripts typically contain 5' caps and 3' poly(A) tails that lack the protein-coding capacity owing to the absence of open reading frames for translation. lncRNAs are considered diagnostic and prognostic biomarkers of malignant solid tumors. By modulating SOX4, STAT3-mediated overexpression of HOXD-AS1 as a ceRNA promotes liver cancer spread [16]. lncRNA MCM3AP-AS1 supports the growth of HCC by targeting the miR-194-5p/FOXA1 axis [17]. This finding implies the important role of lncRNAs in tumor progression, although their exact roles remain unknown.

In the present study, we discovered a novel lncRNA, RP11-620J15.3, which was found to be highly elevated in HCC and seemed to influence the prognosis of HCC patients. RNA-seq differential Gene Set Enrichment Analysis (GSEA) and metabolomics analysis revealed the significant enrichment of RP11-620J15.3 in the glycolytic pathway. Next, we established several HCC cell lines and nude mouse xenograft models with different expression states of RP11-620J15.3.3 to determine whether RP11-620J15.3 was involved in the promotion of glucose metabolism in HCC. As a key oncogene involved in HCC development, RP11-620J15.3 promotes the proliferation and metastatic ability of HCC cells. Subsequently, the molecular mechanism through which RP11-620J15.3 enhances glucose metabolism in HCC was determined by using a luciferase reporter, RNA-seq, and RNA immunoprecipitation (RIP) assays. We found that RP11-620J15.35 promoted glycolysis by sponging the miR-326 transcription levels of the key glucose-6-phosphate isomerase (GPI) gene. Finally, we found that the transcription factor TBP promoted the increased expression of RP11-620J15.3 through the luciferase reporter gene and chromatin immunoprecipitation (ChIP) assays. The present results demonstrated that RP11-620J15.3 promotes glycolytic reprogramming and HCC progression through previously unrecognized regulatory mechanisms. We believe that such information may facilitate the designing of HCC-related therapeutic strategies.

## Materials and methods

### Tissue sampling

At the Zhengzhou University's People's Hospital (Henan, China), 80 pairs of HCC tissues and matched nontumor tissues were collected from HCC patients after obtaining their signed informed consent. None of the patients in this research underwent preoperative radiation or chemotherapy. The Ethics Committee approved this study and it was conducted in compliance with the Declaration of Helsinki (2022NO.33).

### Bioinformatics analysis

In this study, we employed the TCGA dataset to obtain data on HCC patients. For gene enrichment analysis, we used the GSEA (v3.0) software. Additional file 1: Table S1 contains a list of all relevant online analytical websites’ URLs.

### Cell culture

The Cell Bank of the Chinese Academy of Sciences (Shanghai, China) was the source of the original procurement of human HCC cells (i.e., Hep3B, HepG2, Hep-G2, HCC-LM3, Huh-7, and Li7). Fuxiang Biotechnology Company (Shanghai, China) was the source of the initial acquisition of normal liver cells (QSG-7701). The cell lines Hep-G2, HCC-LM3, and Huh-7 were cultured in Dulbecco's modified Eagle’s medium (DMEM) media supplemented with 10% fetal bovine serum (FBS). MEM media supplemented with 10% FBS, 1% non-essential amino acids, and 1% sodium pyruvate was used to cultivate Hep-3B cell lines. MEM medium supplemented with 10% FBS was used to cultivate Li-7 cell lines. The QSG-7701 cell line was cultured in MEM medium supplemented with 10% FBS. All cell lines were incubated at 37 °C in an incubator under a 5% CO_2_ atmosphere.

### RNA extraction, nucleocytoplasmic RNA fractionation analysis, and real-time quantitative PCR (qRT-PCR)

Total RNA was extracted with TRIzol (T9108, Takara, Dalian, China), and reverse transcription was performed by using an enzyme kit. Then, qRT-PCR was performed with the 2ChamQ Universal SYBR qPCR Master Mix (Q711-02, Vazyme, Nanjing, China). Additional file 1: Table S2 lists all PCR primer sequences used in the study. The cytoplasmic and nuclear RNAs used in qRT-PCR reverse transcription were purified and extracted by using the Nuclear and Cytoplasmic RNA Purification Kit (NGB-21000, Norgen, Belmont, CA).

### Western blotting

RIPA buffer (R0010, Solarbio, China) was used to extract all proteins. The relevant antibodies were then treated with the protein extracts transferred onto polyvinylidene difluoride (PVDF) membranes. Additional file 1: Table S3 lists all antibodies used in the study. To determine the ECL signals, the Tanon-5200 Imaging System (Shanghai, China) was employed.

### Plasmids, shRNA transduction, and siRNA transfection

Using Lipo3000 and the pcDNA 3.1-RP11-620J15 plasmid, the overexpressing RP11-620J15 HCC cells were cotransfected. 3. GenMuteTM was used to deliver the siRNA against TATA-box binding protein (TBP) into HCC cells after it was designed and generated by the Qingke company (SL100568, SignaGen, USA). Lentiviruses with shRNA or a negative control construct were packaged by Gene Create Co. (Zhengzhou, China). HCC cells were treated with puromycin at the dosage of 2 g/mL to create cell lines with a stable RP11-620J15.3 knockdown.

### In vivo* metastasis model and xenograft model in nude mice*

Five-week-old male BALB/c nude mice were sourced from Zhengzhou University's Laboratory Animal Center (Zhengzhou, China). RP11-620J15 was transfected into HCC-LM3 cells. 3-sh2, mimics of miR-326, and RP11-620J15. RP11-620J15 or 3-sh2 + miR-326 inhibitors. These mice were subcutaneously injected with the 3-sh2 + GPI plasmid into their left axillary and tail veins. After 20 days of the first collection, subcutaneous tumor samples were collected, while the in vivo metastasis samples were collected after 6 weeks. The Zhengzhou University Center for Animal Experiment reviewed and approved all animal experimentation procedures performed in this study. All experiments were conducted in accordance with the Chinese Animal Welfare Committee's Guidelines for the Care and Use of Laboratory Animals.

### Oncology functional assays

Cell Counting Kit-8 (CCK-8) and colony formation tests were performed to assess the extent of cell proliferation, as described elsewhere [18]. Cell migration was assessed by a wound-healing test, while cell invasion was assessed by a Transwell invasion test [18].

### Construction of reporter plasmids and luciferase reporter detection

The wild-type (WT) and mutant (MUT) binding site sequences for miR-326, TBP, and the RP11-620J15.3 promoter region were introduced into the pGL3 basic vector. The resulting constructs were named the RP11-620J15.3-promoter-WT/Mut, GPI-3'-UTR-WT/Mut, and RP11-620J15.3-WT/Mut. After 48 h of Lipofectamine 3000 transfection, the luciferase activity was measured by using the Dual Luciferase® Reporter Assay System (E1910, Promega, Shanghai, China).

### ChIP assay

ChIP was performed to examine any potential interactions between TBP and the promoter regions of RP11-620J15.3 in HCC cells [18]. The cells were sonicated to a length of 200–1000 bp and crosslinked with formaldehyde. Both an IgG control and an anti-TBP antibody were used throughout the immunoprecipitation procedure. The relative enrichment of the target genes was evaluated by qRT-PCR analysis. A list of all primers and antibody sequences used in the study is given in Additional file 1: Table S2.

### RIP assay

Protein–RNA interactions were identified through the RIP assay. Utilizing the Pierce™ Magnetic RNA–Protein Pull-Down Kit, RIP analysis was carried out (20,164, Thermo Fisher, Shanghai, China). The cells were gathered and thoroughly lysed using RIP lysis buffer in accordance with the manufacturer's instructions. Next, anti-mouse IgG negative control or Ago2 antibody-coated magnetic beads were used to treat RIP Lysate. RNA bound to magnetic beads was extracted, and qRT-PCR was utilized to verify it [18].

### *Fluorescence *in situ* hybridization (FISH)*

AXL-bio (Guangzhou, China) manufactured and designed the RP11-620J15.3 probe. The cells were treated as described previously. The FluoView™ FV1000 confocal microscope (Olympus, Hamburg, Germany) was used to observe RP11-620J15.3 [18].

### RNA-seq

RNA integrity was evaluated using the Agilent 2100. The sequencing libraries were created using the VAHTS mRNA-seq v2 Library Prep Kit (NR602-01, Vazyme) for Illumina. In addition, the Illumina NovaSeq platform was used to sequence the libraries. *P* = 0.05 and an absolute log_2_ fold change > 1 was used to indicate differentially expressed genes.

### Metabolic assays

The cells were plated in a 6-well plate and cultured for 24 h before removing the culture media, after which the glucose and lactate levels were measured using kits (F006-1-1 and A019-2-1, respectively) from the Jiancheng Bioengineering Institute in Nanjing, China. The cells were collected, and the ATP test kit (A095-1-1, Jiancheng Company, China) was used in accordance with the manufacturer's instructions. Protein concentration was used to standardize the results after three independent tests.

The rate of metabolic flux was determined using the Seahorse XFp extracellular flux analyzer (Agilent Technologies, Santa Clara, CA, USA). The cells were planted in Seahorse XFp cell culture plates and allowed to adhere overnight. Then, a medium that was limited by the substrate was used for 16 h. Briefly, for ECAR, the cells were planted at a density of 4 × 10^4^/well in a medium supplemented with 2 mM glutamine. An analyzer was used for measurements in accordance with the manufacturer's instructions after averaging the temperature and pH.

### Statistical analysis

The data were analyzed using Prism 8.2 (GraphPad Software, La Jolla, USA) and SPSS 25.0 (IBM SPSS Statistics, version 25.0). The Student's *t*-test was performed to compare the two groups, and the Kaplan–Meier method was applied for survival analysis. A two-sided unpaired t-test was performed to examine the outcomes of the xenograft growth experiment. To evaluate associations between the gene expressions, Pearson’s correlational analysis was performed. The variance was comparable among the groups that were statistically compared, and all data were consistent with the tests’ presumptions. After three iterations, *p* < 0.05 was considered to indicate a statistically significant difference for each trial.

## Results

### LncRNAs are upregulated in HCC samples and cells

**Fig. 1:**
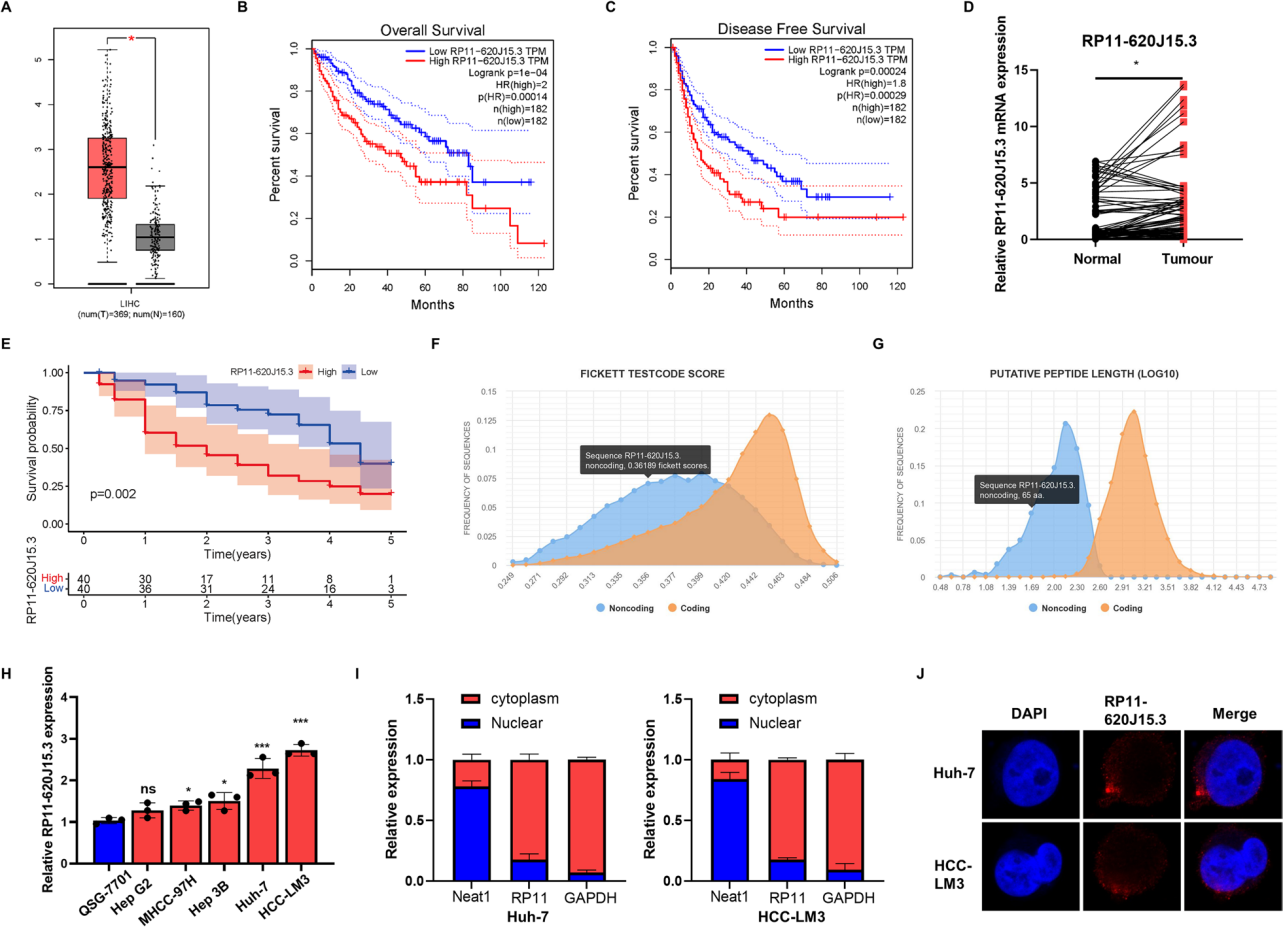
RP11-620J15.3 expression is significantly increased in HCC. **A** HCC and non-tumor tissue RP11-620J15.3 transcription levels based on the analysis of the TCGA-LIHC dataset.** B** Overall survival curves of HCC patients based on the RP11-620J15.3 expression. **C** Disease-free curves of HCC patients based on the RP11-620J15.3 expression. **D** The expression of RP11-620J15.3 in 80 pairs of HCC tissues and their paired non-tumor tissues.** E** A total of 80 cases of HCC patients were based on the overall survival curve of the RP11-620J15.3 expression. **F, G** The coding possibility of RP11-620J15.3 was analyzed by CPAT and CPT. **H** The RP11-620J15.3 expression in hepatocytes and HCC cell lines. **I, J** The main position of RP11-620J15.3 was analyzed by the nuclear-cytoplasmic RNA fractionation and FISH experiments. *p < 0.05, **p < 0.01, ***p < 0.001

To explore the role of HCC-related lncRNAs, we identified a novel lncRNA, namely RP11-620J15.3 (chromosome 12: 58,325,311-58,329,958). A high expression of RP11-620J15.3 in the HCC tissues has been associated with a poor prognosis (Fig. 1A–C). At the People's Hospital of Zhengzhou University, 80 pairs of HCC tissues with matched neighboring noncancerous tissues were collected and subjected to qRT-PCR, and clinical data were also examined (Fig. 1D, E). Univariate Cox regression analysis was performed to assess whether the RP11-620J15.3 expression could serve as an independent risk factor for the poor prognosis of HCC patients. Furthermore, UCSC analysis revealed that RP11-620J15.3 was conserved, and CPAT and CPC2 revealed that RP11-620J15.3 possessed noncoding characteristics (Fig. 1F–G). Next, we detected the RP11-620J15.3 expression in HepG2, Hep3B, Huh7, and LM3 HCC cell lines and normal human hepatocyte QSG-7701 cells. The results implied that the RP11-620J15.3 expression was increased in HCC cells when compared with that in the LO2 cells, and the highest levels of expression were noted in the Huh7 and LM3 cell lines (Fig. 1H). Nuclear cytoplasmic RNA isolation and FISH analysis were performed to analyze the subcellular localization of RP11-620J15.3 in HCC cells, and the results suggested that RP11-620J15.3 was mainly located in the cytoplasm (Fig. 1I, J). These findings implied that RP11-620J15.3 was elevated in HCC tissues and could have possibly contributed to HCC progression.

### *RP11-620J15.3 promotes HCC progression *in vivo* and *in vitro

**Fig. 2:**
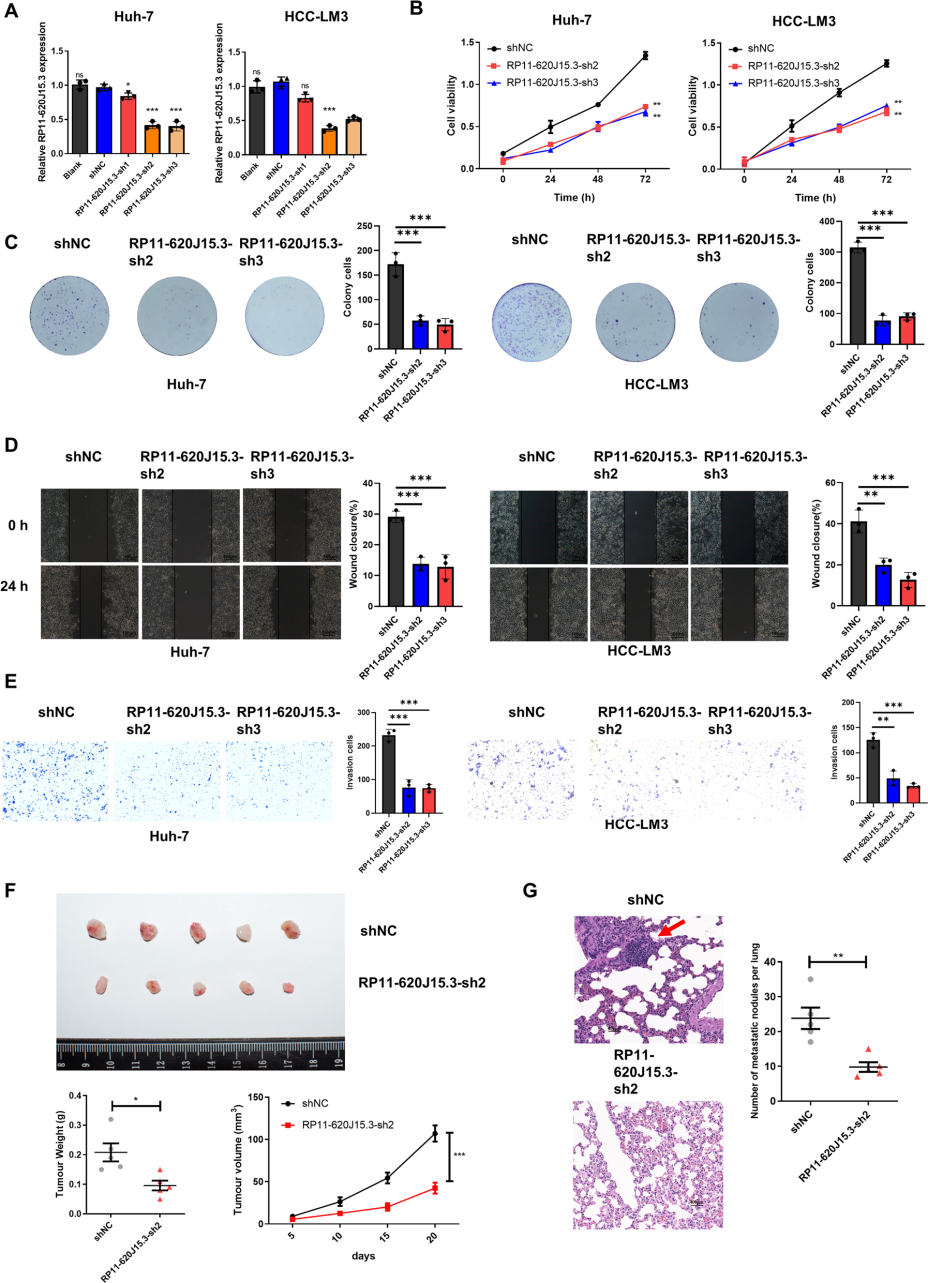
RP11-620J15.3 promotes HCC proliferation and metastasis. **A** Expression of RP11-620J15.3 in Huh-7 and HCC-LM3 cells transfected with shNC and RP11-620J15.3 targeting shRNA. **B, C** CCK-8 and colony formation assay demonstrated the proliferation ability of Huh-7 and HCC-LM3 cells. **D** Scratch wound-healing assay revealed the cell-migration ability. **E** Transwell Matrigel invasion assay demonstrated cell-invasion ability. **F** A xenograft model was established from control or stable RP11-620J15.3 knockdown HCC-LM3 cells and used to evaluate the weight of the anatomical tumor. **G** The metastasis in the lungs of nude mice injected with shNC or RP11-620J15.3-sh2 HCC cells. Magnification, 100 × . *p < 0.05, **p < 0.01, ***p < 0.001

We created HCC-LM3 and Huh-7 cells with stable knockdowns of RP11-620J15.3 and verified the knockdown by qRT-PCR. These cell lines were selected for further analysis as they exhibited high expressions of RP11-620J15.3 (Fig. 2A). CCK-8 and colony formation assays revealed that the silencing of RP11-620J15.3 significantly reduced the proliferation and development of HCC cells (Fig. 2B, C). The findings of the transwell and wound healing assays demonstrated that the inhibition of RP11-620J15.3 knockdown prevented HCC cell invasion and migration (Fig. 2D, E).

We created HCC-LM3 cells with stable RP11-620J15.3 knockouts and subcutaneously implanted HCC cells into nude mice to further investigate the role of RP11-620J15.3 in tumor formation in vivo. The low tumor volume and weight in the RP11-620J15.3 expression group were dramatically reduced when compared to that in the control group (Fig. 2F). Furthermore, we constructed a metastatic model via tail vein injection, and RP11-620J15.3 deletion resulted in fewer lung metastatic nodules (Fig. 2G). In summary, RP11-620J15.3 promoted HCC proliferation and metastasis both *in vivo* and *in vitro*.

### Knockdown of RP11-620J15.3 inhibits the Warburg effect in HCC cells

**Fig. 3:**
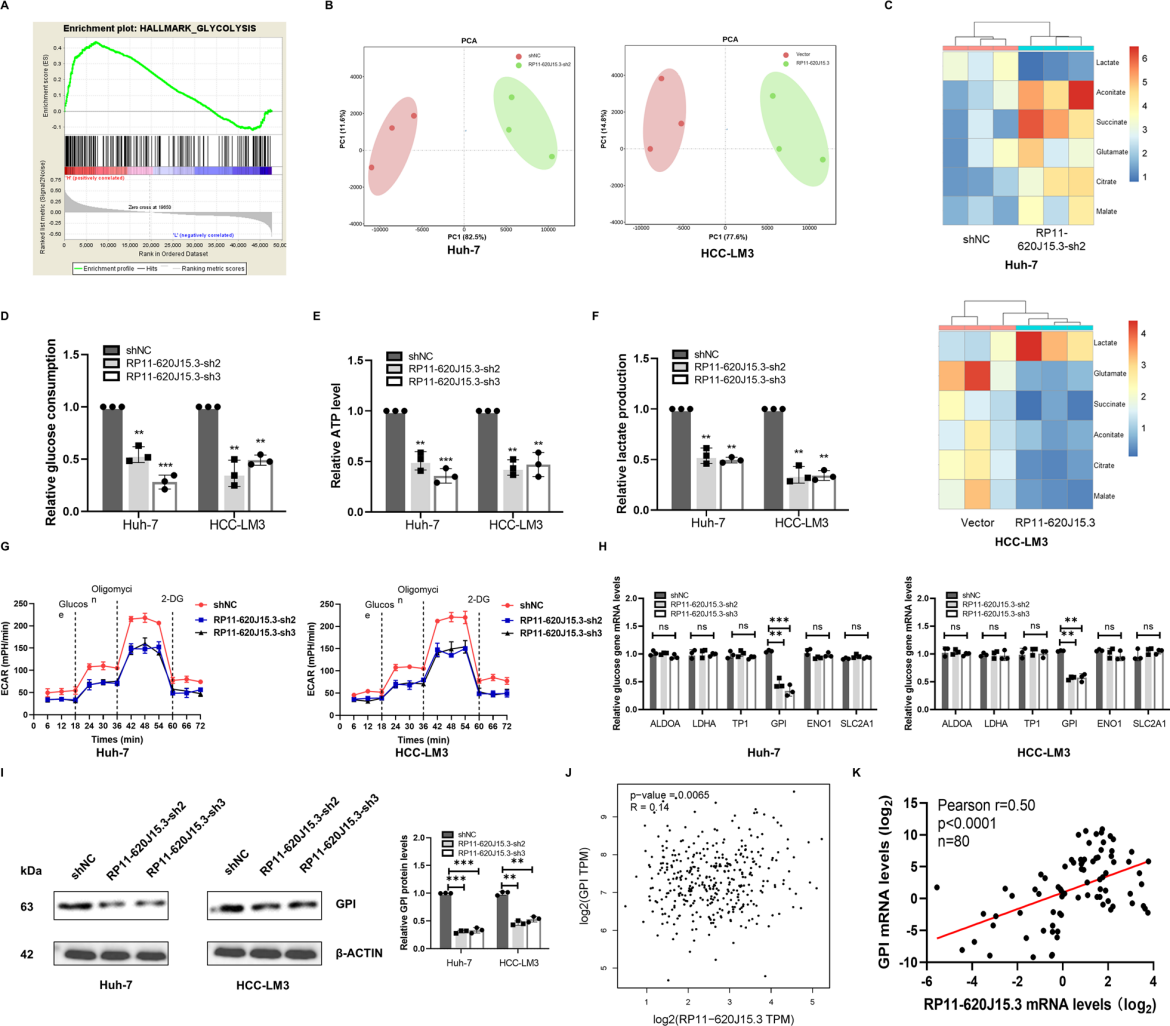
Knockdown of RP11-620J15.3 inhibits glycolysis in HCC. **A** Positive enrichment for the gene signature associated with fatty acid metabolism between the low and high RP11-620J15.3 expression groups of the RNA-seq dataset. **B** Nontargeted metabolomics GC–MS analysis of glycolysis and TCA cycle metabolites in HCC cells. **C** Heatmap displaying changes in the metabolites of glycolysis or OXPHOS. **D–F** Changes in the glycolysis-related metabolites of HCC cells. **G** Changes in the ECAR rate of HCC cells. **H, I** In the HCC cells with RP11-620J15.3 knockdown, the mRNA and protein levels of the related lipogenic enzymes were significantly reduced. **J, K** Pearson’s correlational analysis of RP11-620J15.3 and GPI in the TCGA-LIHC dataset and 80 pairs of tissues from Zhengzhou University. *p < 0.05, **p < 0.01, ***p < 0.001

**Fig. 4:**
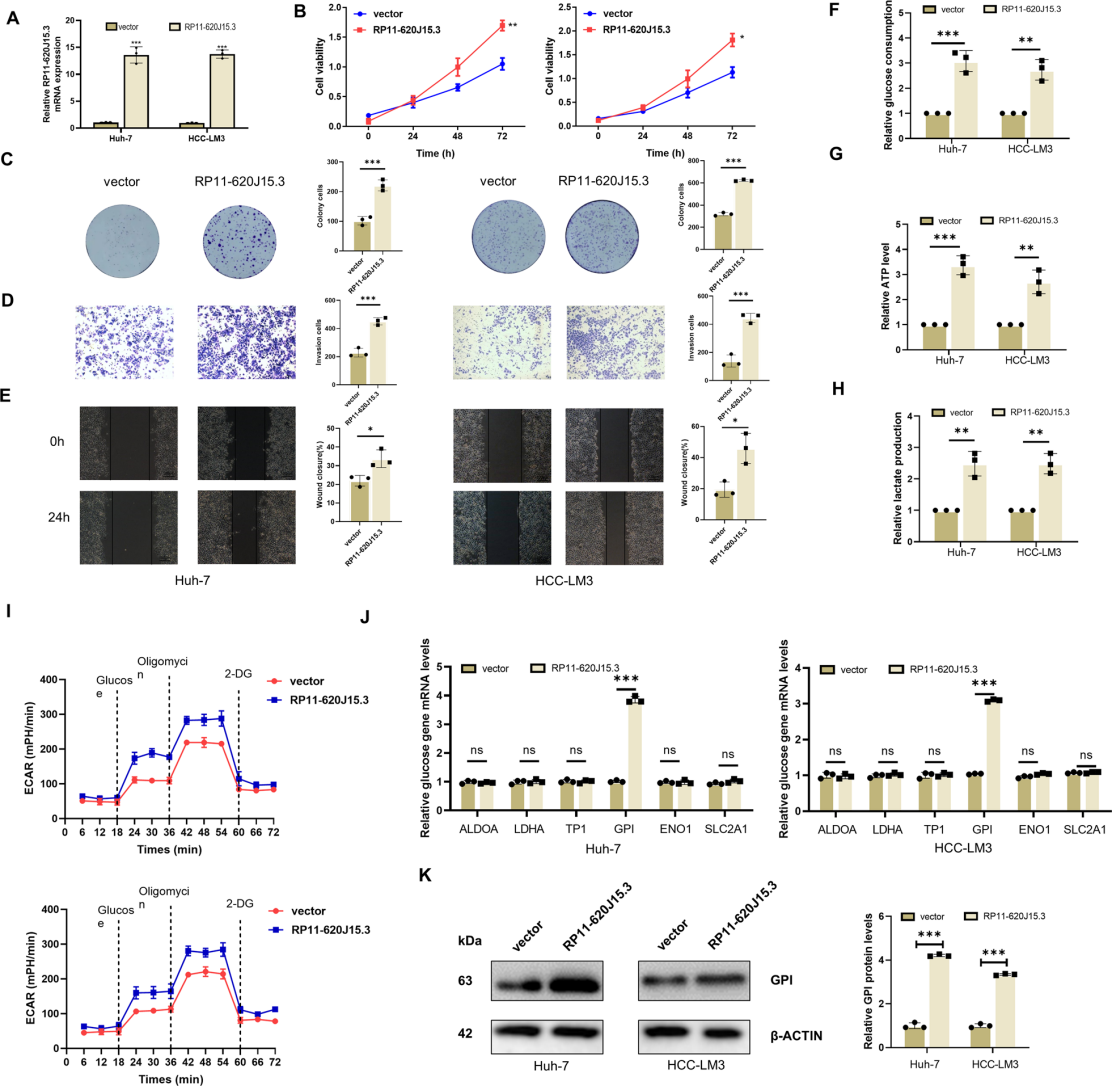
Overexpression of RP11-620J15.3 promotes glycolysis and progression in HCC. **A** The expression of RP11-620J15.3 in Huh-7 and HCC-LM3 cells transfected with RP11-620J15.3 plasmid. **B, C** CCK-8 and colony-formation assay demonstrated the proliferation ability of Huh-7 and HCC-LM3 cells. **D, E** Scratch wound-healing assay and Transwell invasion assay showed the migration and invasion abilities of the cells. **F–H** Changes in the glycolysis-related metabolites in HCC cells overexpressing RP11-620J15.3. **I** Changes in the ECAR rate in HCC cells overexpressing RP11-620J15.3. **J, K** The mRNA and protein expression of the related lipogenic enzymes in Huh7 and HCC-LM3 cells overexpressing RP11-620J15.3. *p < 0.05, **p < 0.01, ***p < 0.001

To determine the potential molecular mechanism by which RP11-620J15.3 affects HCC growth, we detected transcript changes after RP11-620J15.3 knockdown through RNA-seq and performed GSEA using RNA-seq data. Genes involved in the glycolysis pathway were found to be significantly upregulated, and GPI was the most significantly upregulated one (Fig. 3A). Principal component analysis (PCA) of metabolomics demonstrated the relatedness of samples (Fig. 4B). Heatmap displayed differentially expressed metabolites of the glucose metabolism pathways (Fig. 4C). To further systematically analyze whether RP11-620J15.3 induces the Warburg effect in HCC cells, we tested the glucose uptake and lactic acid production ability after RP11-620J15.3 was knocked out. When compared with the control cells, the glucose uptake, ATP production, and lactic acid production were reduced in the RP11-620J15.3-silenced cells (Fig. 3D–F). Next, we detected the extracellular acidification rate (ECAR) of HCC cells. Moreover, RP11-620J15.3 knockdown significantly reduced the ECAR level in the HCC cell lines (Fig. 3G). Subsequently, we detected the differentially expressed genes related to glycolysis by qRT‒PCR and Western blotting. The results suggested that RP11-620J15.3 knockdown in HCC cells significantly inhibited GPI mRNA and protein expression, whereas no significant changes were recorded in other glycolytic genes, including *LDHA*, *ALDOA*, *ENO1*, *TPI1*, and *SLC2A1* (Fig. 3H–I). In addition, we assessed data from the TCGA-LIHC database, and the results indicated that RP11-620J15.3 was positively correlated with GPI (Fig. 3J). RP11-620J15.3 and GPI correlation analyses in 80 HCC samples revealed that RP11-620J15.3 was significantly positively correlated with GPI (r = 0.50, *p* < 0.0001) (Fig. 3K). These results indicated that RP11-620J15.3 enhances the Warburg effect of HCC cells through GPI.

### RP11-620J15.3 overexpression enhances proliferation, metastasis, and the Warburg effect of HCC cells

To further verify the oncological function of RP11-620J15.3, we designed plasmids to construct HCC-LM3 and Huh-7-cell lines overexpressing RP11-620J15.3, and the expressions were verified by qRT‒PCR (Fig. 4A). Consistent with the results obtained in the context of RP11-620J15.3 knockdown, CCK-8 assay, and plate-cloning experiments revealed that RP11-620J15.3 overexpression significantly increased HCC proliferation and growth cells (Fig. 4B–C). Then, Transwell and cell scratch analysis revealed that RP11-620J15.3 overexpression significantly upregulated HCC cell migration and invasion (Fig. 4D–E). When compared with the control cells, the cells overexpressing RP11-620J15.3 showed increased glucose uptake, ATP production, and lactic acid production (Fig. 4F–H). Next, we detected the ECAR of HCC cells. RP11-620J15.3 overexpression significantly increased the ECAR level in HCC cell lines (Fig. 4I). In addition, RP11-620J15.3 overexpression significantly increased GPI mRNA and protein expressions (Fig. 4J–K).

### RP11-620J15.3 functions as a sponge to negatively regulate the miR-326 expression

**Fig. 5:**
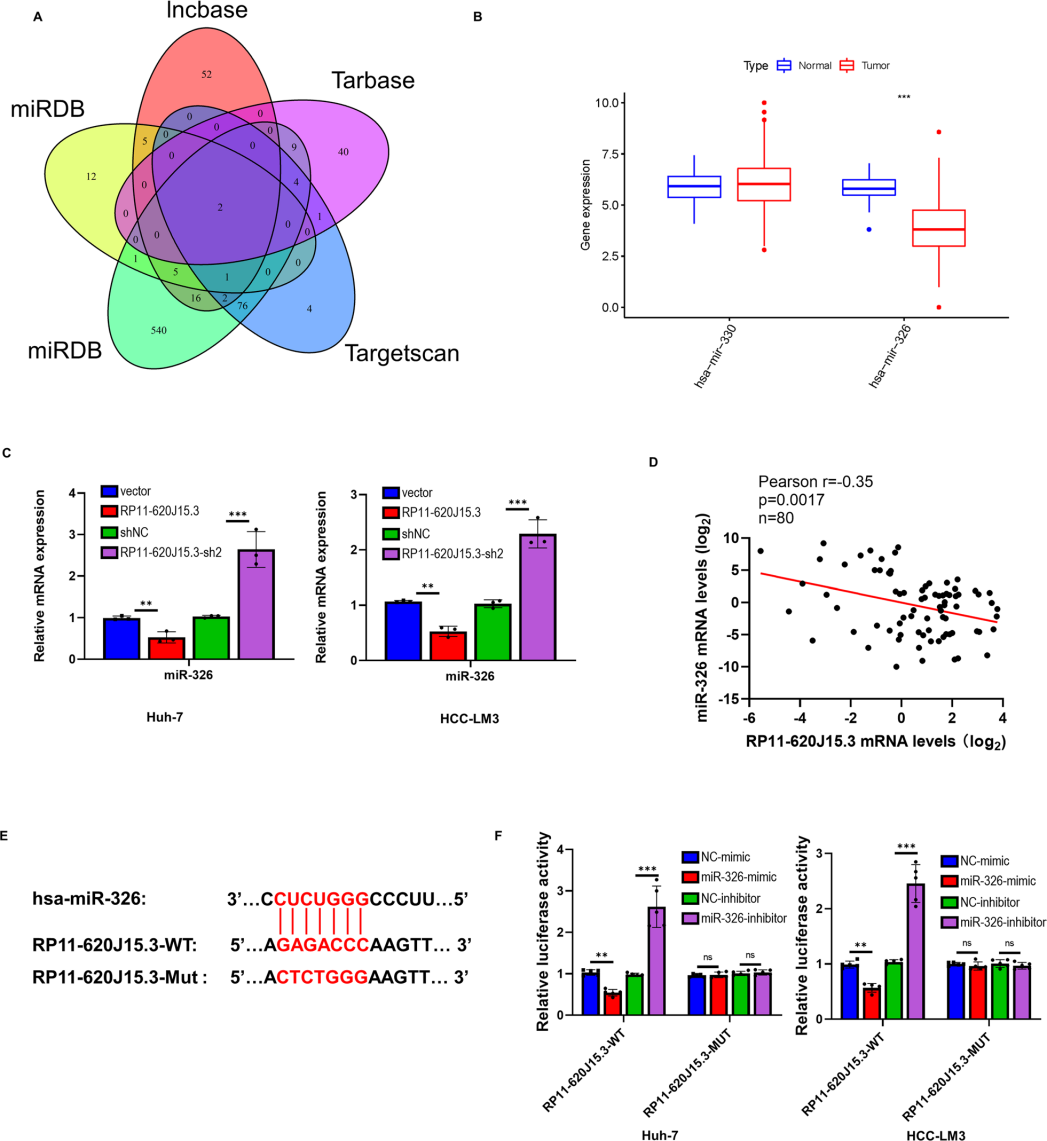
RP11-620J15.3 directly binds to miR-326 and negatively regulates the miR-326 expression. **A** Bioinformatics analysis of the potential target genes was performed by using prediction software (such as mirdb, lncbase, tarbase, and targetscan). **B** The target miRNA expression in HCC tumors and non-tumor tissues in the TCGA database. **C** qRT-PCR results showed the miR-326 expression in RP11-620J15.3 overexpressing or knockdown HCC cells. **D** Pearson’s correlational analysis of RP11-620J15.3 and miR-326 in 80 HCC tissue samples. **E** Schematic diagram of the potential binding site between RP11-620J15.3 and miR-326. **F** Dual luciferase reporter gene detection confirmed the interaction between RP11-620J15.3 and miR-326. *p < 0.05, **p < 0.01, ***p < 0.001

There are numerous mechanisms through which lncRNAs control cellular functions, and the majority of lncRNAs serve as molecular scavengers for miRNAs. To control GPI production and functions in HCC, we, therefore, postulated that RP11-620J15.3 may interact with miRNA as a ceRNA. The potential miRNAs that RP11-620J15.3 and GPI may interact with were predicted by using the bioinformatics tools miRDB, Incbase, and TargetScan. These findings indicated that two miRNAs (miR-330 and miR-326) may be under the control of RP11-620J15.3 (Fig. 5A). We discovered that only miR-326 was expressed at low levels in HCC with reference to the TCGA database (Fig. 5B). In HCC-LM3 and Huh-7-cell lines with RP11-620J15.3 knockdown, the miR-326 expression was significantly increased, whereas the RP11-620J15.3 overexpression yielded the opposite results (Fig. 5C). Our qPCR analysis of 80 HCC and paired adjacent tissues revealed that miR-326 was significantly negatively correlated with the RP11-498C9.1 expression (Fig. 5D). To further confirm the interaction between RP11-620J15.3 and miR-326, we constructed WT and mutant RP11-620J15.3 constructs, overexpressed miRNA with RNA mimics, and inhibited the miRNA expression in HCC cells using miRNA inhibitors (Fig. 5E). The relative luciferase activity of the RP11-620J15.3-WT construct was significantly decreased in the miR-326 simulation group than in the NC group, albeit it significantly increased in the miR-326 inhibitor group, according to the results of the twofold luciferase reporter gene detection (Fig. 5F). No significant differences were noted in the relative luciferase activity of the RP11-620J15.3-MUT construct between the inhibitor and NC groups (Fig. 5F). These data indicate that RP11-620J15.3 acts as a miR-326 sponge in HCC cells.

### RP11-620J15.3 and miR-326 regulate the GPI expression as ceRNAs

**Fig. 6:**
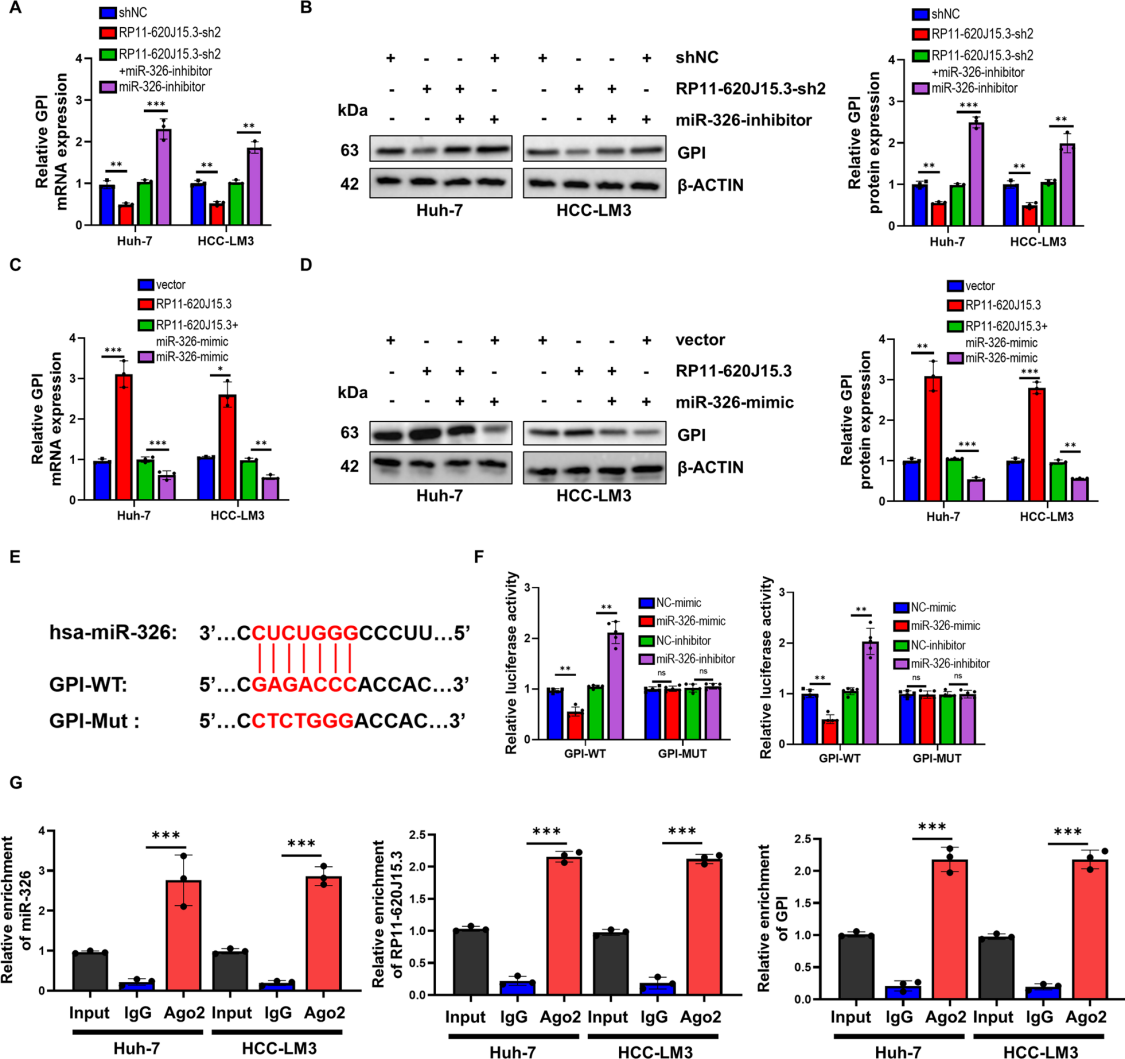
RP11-620J15.3 and miR-326 directly bind to GPI and regulates GPI expression. **A–D** GPI mRNA and protein expression in co-transfected HCC cells. **E** The schematic representation of the potential binding sites between miR-326 and GPI. **F, G** The dual luciferase reporter gene and RIP test confirmed the interaction among RP11-620J15.3, miR-326, and GPI. *p < 0.05, **p < 0.01, ***p < 0.001

To confirm the relationship among RP11-620J15.3, miR-326, and GPI, we demonstrated that transfection of miR-326 mimics or inhibitors significantly weakened the effects of RP11-620J15.3 upregulation or downregulation on the GPI mRNA and protein levels by qPCR and Western blotting (Fig. 6A–D). To further confirm the direct interaction between GPI mRNA and the 3'UTR complementary binding site in miR-326, we constructed a luciferase reporter vector containing GPI mRNA (GPI-WT) with either the WT 3'UTR or a mutant 3'UTR sequence without the miR-326 complementary binding site (GPI-MUT; Fig. 6E). We found that, when compared with the negative control group of HCC cells transfected with GPI-WT, the transfected miR-326 analog or inhibitor significantly reduced or increased the luciferase activity, respectively (Fig. 6F). However, transfection of miR-326 mimics or inhibitors did not affect the luciferase activity in GPI-MUT-transfected HCC cells (Fig. 6F). These results indicated that miR-326 inhibits the GPI expression by binding to the 3'UTR of GPI. Finally, the Ago2 RIP experiment revealed that RP11-620J15.3, miR-326, and GPI were precipitated by Ago2 (Fig. 6G). These findings indicate that RP11-620J15.3 acts as a ceRNA and promotes the GPI expression by acting as a miR-326 sponge in HCC cells.

### RP11-620J15.3 regulates HCC cell proliferation, transformation, and glycolytic activity by regulating the miR-326/GPI axis

**Fig. 7:**
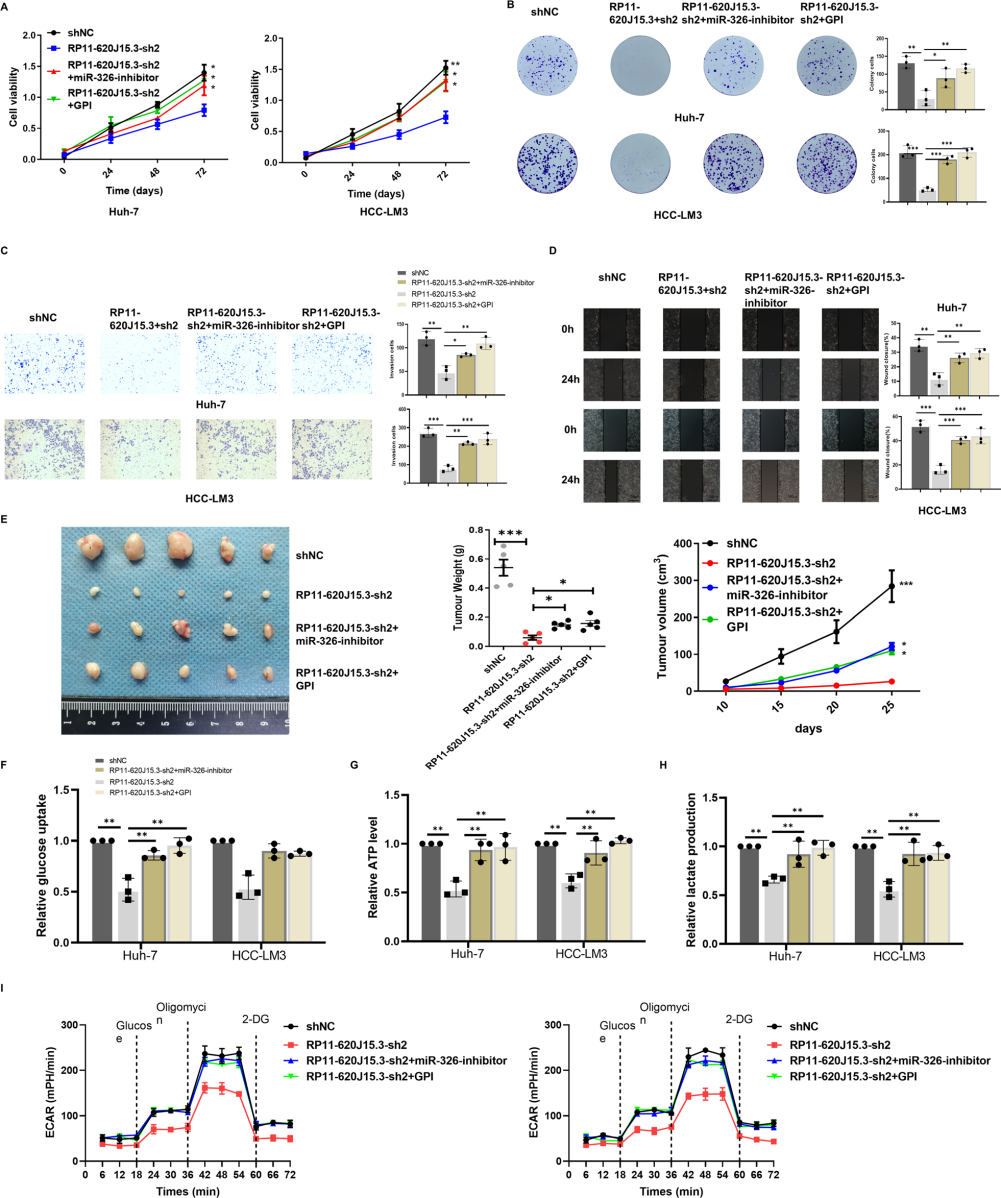
RP11-620J15.3 promotes cell growth and metastasis and glycolysis in HCC through the miR-326/GPI axis. **A, B** The proliferation ability of HCC cells was assessed by CCK-8 and colony-formation assays. **C** The wound healing assay revealed the migration ability of HCC cells. **D** The Transwell invasion assay with Matrigel indicated cell-invasion ability. **E** The tumor growth curves from the xenograft model established with co-transfected HCC-LM3 cells and evaluation of the weights of the excised tumors. **F–H** Changes in the glycolysis-related metabolites in co-transfected HCC cells. **I** Changes in the ECAR rate in co-transfected HCC cells. *p < 0.05, **p < 0.01, ***p < 0.001

To determine whether the RP11-620J15.3/miR-326/GPI axis is the key pathway regulating HCC cell proliferation, transformation, and glycolytic activity, we transfected miR-326 inhibitors or plasmids containing GPI mRNA into Huh-7/LM3 cells with RP11-620J15.3 knockdown. CCK-8 and plate cloning experiments revealed that the inhibition of miR-326 or GPI overexpression prevented the reduction in HCC cell growth and proliferation caused by RP11-620J15.3 knockdown (Fig. 7A–B). Transwell and cell scratch analysis results indicated that HCC cell migration and invasion exhibited the same trend (Fig. 7C–D). To further explore the role of the RP11-620J15.3/miR-326/GPI axis in tumor growth in vivo, we subcutaneously injected HCC-LM3 cell lines transfected with miR-326 inhibitors or plasmids containing GPI mRNA that stably knocked out RP11-620J15.3 into BALB/c nude mice. Our results indicated that the inhibition of miR-326 or GPI overexpression rescued the reduction in tumor volume and weight induced by the low expression of RP11-620J15.3 (Fig. 7E). In conclusion, both in vivo and in vitro, the RP11-620J15.3/miR-326/GPI axis supports HCC proliferation and metastasis. We further explored whether RP11-620J15.3 enhances glycolysis in HCC cells by sponging miR-326. We found that after a miR-326 inhibitor or a plasmid containing GPI mRNA was transfected into HCC cells with RP11-620J15.3 knockdown, the glucose uptake, lactic acid production, and ATP production were significantly increased (Fig. 7F–H). Next, we discovered that RP11-620J15 was responsible for the decrease in ECAR. In fact, the MiR-326 inhibitor or transfection with a plasmid carrying GPI mRNA could restore the effect of RP11-620J15.3 knockdown (Fig. I). These results showed that the RP11-620J15.3/miR-326/GPI axis promoted HCC proliferation and transformation in vivo and in vitro and promoted glycolysis in HCC cells.

### TBP is a transcription factor of RP11-620J15.3

**Fig. 8:**
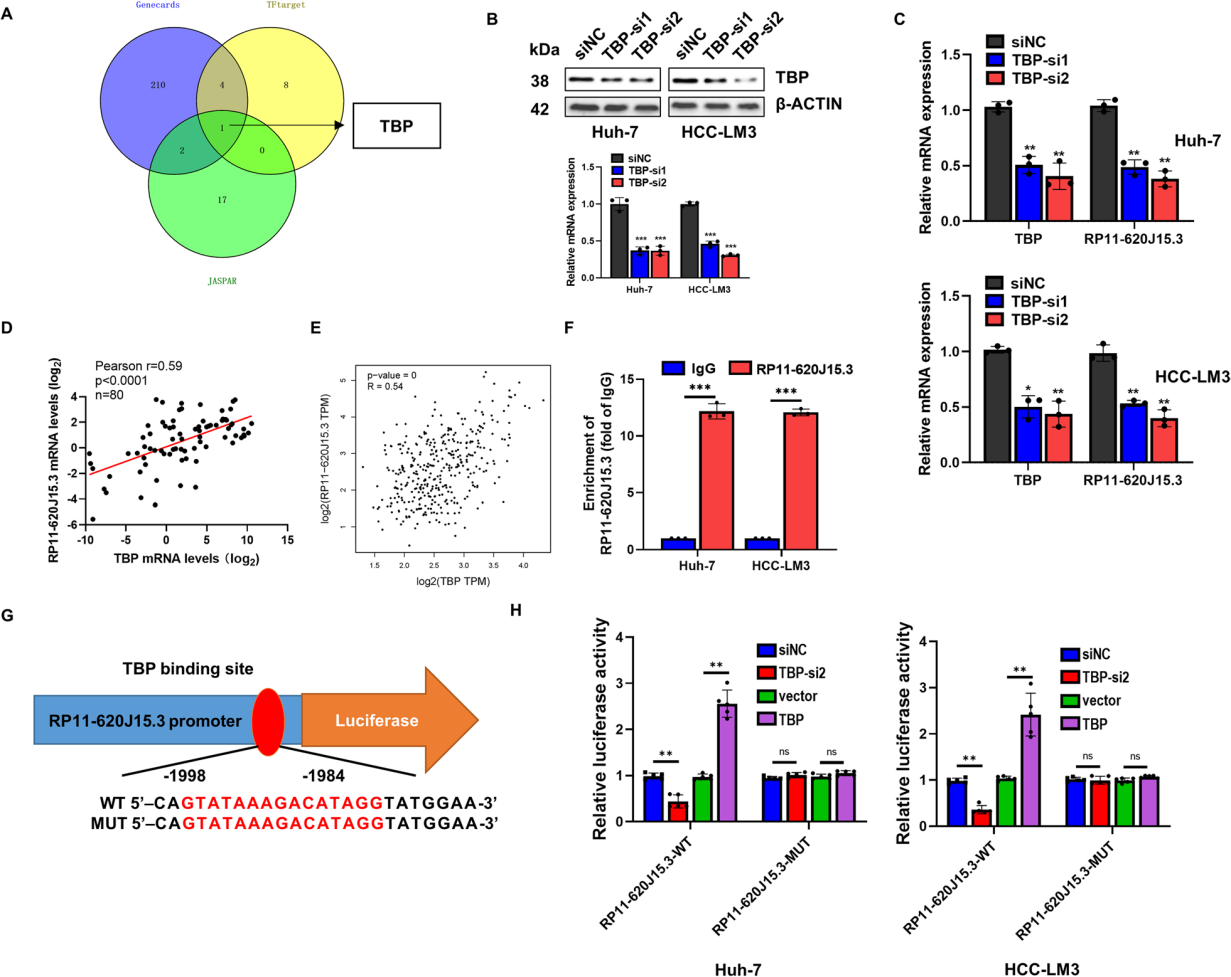
RP11-620J15.3 is directly regulated by the transcription factor TBP. **A** Transcription factors that may regulate the transcription of RP11-620J15.3 were identified by prediction databases (tfTarget, GeneCards, and JASPAR). **B, C** The expression levels of TBP and RP11-620J15.3 in HCC cells with TBP knockdown were verified by qRT-PCR and WB. **D, E** Pearson’s correlational analysis of RP11-620J15.3 and TBP in the TCGA-LIHC dataset and 80 pairs of tissues from Zhengzhou University. **F** The CHIP assay results confirmed the direct binding between TBP and the RP11-620J15.3 promoter region. **G** The binding sequence of TBP and RP11-620J15.3 was predicted from the JASPAR database. *p < 0.05, **p < 0.01, ***p < 0.001

Finally, using geneCARDS, hTFtarget, and UCSC, we predicted that TBP may be a transcription factor regulating the RP11-620J15.3 expression, which clarified the reason for RP11-620J15.3 overexpression in HCC (Fig. 8A). We created a sequence to inhibit TBP production to confirm whether TBP is a transcription factor of RP11-620J15.3. Our findings thus demonstrated that TBP knockdown drastically decreased the RP11-620J15.3 expression (Fig. 8B–C). In addition, BP was overexpressed in the HCC tissues and had a strong positive correlation with RP11-620J15.3, according to the TCGA data. This conclusion was supported by qPCR data from 80 pairs of HCC and normal tissues (Fig. 8D–F). The ChIP qPCR outcomes further demonstrated that TBP binds directly to the RP11-620J15.3 promoter region (Fig. 8G). We created a reporter plasmid with the potential TBP-binding sites and the RP11-620J15.3 promoter, as predicted by UCSC and JASPAR, and observed the luciferase reporter gene expression in Huh-7 and LM3 cells to confirm that RP11-620J15.3 is regulated by TBP transcription (Fig. 8H). Our findings demonstrated that, in the RP11-620J15.3 promoter WT group, the TBP overexpression or knockdown significantly boosted or decreased the fluorescence activity (Fig. 8H).

## Discussion

lncRNA dysfunction is known to be involved in the occurrence and development of HCC. In the present study, we discovered that RP11-620J15.3 was overexpressed in HCC and was associated with various clinical malignant features, such as tumor size. Moreover, we observed an increase in the RP11-620J15.3 levels in HCC cells. Importantly, our data and TCGA data cumulative suggest that the total survival and disease-free survival of HCC patients with high RP11-620J15.3 levels were significantly reduced. A series of functional experiments revealed that RP11-620J15.3 promoted HCC cell proliferation and glycolytic activities. As such, we identified RP11-620J15.3 as a new carcinogen in HCC.

Metabolic reprogramming is now recognized as a core hallmark of cancer, and it is an evolving area of therapeutic interest [19]. In addition, augmented aerobic glycolysis contributes to the growth, apoptosis resistance, metastasis, immune escape, and drug resistance of HCC [20]. GPI is a commonly expressed housekeeping cytoplasmic enzyme. Apart from its role in carbohydrate metabolism, its overexpression was associated with poor prognosis and reduces the overall survival of cancer [21]. It was demonstrated to contribute to the aggressive phenotype of colorectal cancer, pancreatic cancer, HCC, endometrial cancer, and bone and soft tissue cancer [22]. TCGA database and our data indicated that the RP11-620J15.3 and GPI mRNA levels in HCC tissues were positively correlated. We thus confirmed that RP11-620J15.3 positively regulated the GPI expression in HCC cells, as demonstrated by qRT‒PCR and Western blotting. In addition, RP11-620J15.3 acts as an endogenous competing RNA sponge of miR-326, thus promoting the expression of the miR-326 target gene GPI. Notably, the recovery of the GPI expression eliminated the effects of RP11-620J15.3 knockdown on HCC cell proliferation and glycolytic activity. Our data indicated that GPI played a key role in HCC progression induced by RP11-620J15.3. These findings provide new insights into the regulatory mechanisms involved in the GPI expression of HCC cells.

Cancer is a disease related to genomic instability, often resulting from oncogene activation [23]. However, only little is known about the molecular mechanism of oncogene activation. The transcription factors are the master regulators for several important biological processes including cell development, metabolism, cell proliferation, cell migration, and metastasis [24]. It has been reported that transcription factor TBP stimulates RNA synthesis to promote tumor cell proliferation [25]. Our results demonstrated that TBP directly binds to RP11-620J15.3 and activates the RP11-620J15.3 transcription.

Some widely used drugs are known to target the glycolysis gene to inhibit glycolysis and treat HCC. Although some of these drugs targeting glycolysis are currently undergoing phase I or II clinical trials, they are mostly non-specific inhibitors or activators with numerous adverse effects and poor safety in terms of clinical applications [8]. LncRNA is a new target for treating HCC [26]. In recent years, significant progress has been made in delivery strategies targeting ncRNA targets. Moreover, the lncRNA targe ting systems are simpler and more effective than developing specific activators or inhibitors of proteins [26]. Considering that RP11-620J15.3/miR-326 can regulate the known glycolysis therapeutic target GPI, significant progress is expected to be made in the short term through the currently available simple and easy-to-operate ncRNA delivery systems.

In summary, we discovered RP11-620J15.3 as a brand-new carcinogen in HCC. Through sponging miR-326, RP11-620J15.3 increases GPI transcription and expression, which, in turn, promotes HCC cell proliferation and glycolytic activities. The poor prognosis of HCC is thus linked to the overexpression of RP11-620J15.3. Therefore, potential therapeutic targets and prognostic markers for HCC should be searched for in the RP11-620J15.3-signaling pathway.

## Supplementary Information

**Additional file 1.** Supplementary tables S1–S4.

## Data Availability

The data that support the findings of this study are available from the corresponding author upon reasonable request.

## Abbreviations

AGO2: Argonaute2
ceRNAs: Competing endogenous RNAs
ChIP: Chromatin immunoprecipitation
DMEM: Dulbecco’s modified eagle’s medium
FISH: Fluorescence in situ hybridization
HCC: Hepatocellular carcinoma
RP11-620J15.3: LncRNA hepatocarcinoma lipid reprogramming regulator 1
GPI: Heterogeneous nuclear ribonucleoprotein U
lncRNA: Long noncoding RNA
miRNAs: MicroRNAs
RIP: RNA immunoprecipitation
shRNA: Short hairpin RNA
TCGA-LIHC: The Cancer Genome Atlas-Liver Hepatocellular Carcinoma
