# Supplementary material for: LncRNA RP11-620J15.3 promotes HCC cell proliferation and metastasis by targeting miR-326/GPI to enhance glycolysis
Source: Biol Direct. 2023 Apr 5;18:15. doi: 10.1186/s13062-023-00370-0 (PMC10077620; doi:10.1186/s13062-023-00370-0)
Supplement: Supplementary file 1 — Additional file 1. Supplementary tables S1–S4. [file 13062_2023_370_MOESM1_ESM.pdf]

Supplementary Table S1 Online analysis website

website	URL
GEPIA	http://gepia.cancer-pku.cn/
Ensembl	http://grch37.ensembl.org/index.html
UCSC	http://genome.ucsc.edu/
CPC2	http://cpc2.gao-lab.org/
CPAT	http://lilab.research.bcm.edu/
Mirdb	http://mirdb.org/
LncBase	http://carolina.imis.athena-innovation.gr/diana_tools/
Starbase	http://starbase.sysu.edu.cn/starbase2/index.php
Targetscan	http://www.targetscan.org/vert_72/
TCGA-LIHC	https://portal.gdc.cancer.gov/

Supplementary Table S2 Primer sequences, siRNAs and shRNA used in this study.

Primer name		Sequence (5'-3')
RP11-620J15.3	F	ACGGTGTTCTGGGAGGAGTT
	R	TATCTCTGCCTCTCCCGCTA
miR-326	F	CATCTGTCTGTTGGGCTGGA
	R	AGGAAGGGCCCAGAGGCG
GPI	F	CAAGGACCGCTTCAACCACTT
	R	CCAGGATGGGTGTGTTTGACC
ALDOA	F	CATTCTGGCTGCGGATGAGTCT
	R	CACACGGTCATCAGCACTGAAC
LDHA	F	CTGGGAGTTCACCCATTAAGCT
	R	CAGGCACACTGGAATCTCCAT
TP1	F	AGTGACTAATGGGGCTTTTACTG
	R	GCCCAATCAGCTCATCTGACTC
ENO1	F	ATGTCTATTCTCAAGATCCATGCCAGG
	R	CTACTTGGCCAAGGGGTTTCTGAAG
SLC2A1	F	GGCCAAGAGTGTGCTAAAGAA
	R	ACAGCGTTGATGCCAGACAG
β-Actin	F	ATCATGTTTGAGACCTTCAACA
	R	CATCTCTTGCTCGAAGTCCA
GAPDH	F	CCCATCACCATCTTCCAGGAG
	R	GTTGTCATGGATGACCTTGGC
U6	F	CGCTTCGGCAGCACATATAC
	R	CAGGGGCCATGCTAATCTT
TBP	F	CCGGAATCCCTATCTTTAGTCC
	R	GCCTTTGTTGCTCTTCCAAAAT
RP11-620J15.3-CHIP	F	GTGAAGCGATAAGACGCACG
	R	CGCAGATAACGGTCAATCGC
shNC		ATCTGCCTTCGAGCCACGT
RP11-620J15.3-sh1		AAUAUCUCUGCCUCUCCCGUU
RP11-620J15.3-sh2		UUUAGCACAUUCUCUGUUUUU
RP11-620J15.3-sh3		UGUCUGUAUGUUCUUUAGCUU
siNC		UUCUCCGAACGUGUCACGU
TBP-si1		AAUAUCUCUGCCUCUCCCGUU
TBP-si2		UUUAGCACAUUCUCUGUUUUU

Supplementary Table S3 Primary antibodies used in this study

Antigens	Manufacturer	Catalog Number	Application
GPI	Proteintech	15171-1-AP	1:1000 for WB;
TBP	Proteintech	66166-1-Ig	1:10000 for WB;
β-actin	Proteintech	66009-1-Ig	1:1000 for WB;
IgG	Servicebio	GB23303	IP
IgG	Servicebio	GB23301	IP
AGO-2	Proteintech	66720-1-Ig	IP

Supplementary Table S4 Correlation between clinicopathological features and RP11-620J15.3 expression in HCC tumor tissues.

Characteristics		Number	RP11-620J15.3 expression		P value
			High	Low	
Gender					*0.8217*
	Male	35	17	18	
	Female	45	23	22	
Age					*0.8226*
	<65	37	19	18	
	≥65	43	21	22	
HBV infection					*0.1082*
	No	31	12	19	
	Yes	49	28	21	
AFP					*0.4957*
	≤400 (μg/L)	33	15	18	
	>400 (μg/L)	47	25	22	
Tumor size					***0.01388****
	≤5cm	39	14	25	
	>5cm	41	26	15	
TNM staging					*0.2634*
	I-II	39	17	22	
	III-IV	41	23	18	
Lymph node metastasis					*0.1035*
	Absent	51	22	29	
	Present	29	18	11	
BCLC stage					*0.1119*
	Low	33	13	20	
	High	47	27	20	
PVTT					***0.04252****
	NO	45	18	27	
	YES	35	22	13	

*The expression of RP11-620J15.3 were compared between the tumor tissue and the normal tissue. BCLC, Barcelona Clinic Liver Cancer; PVTT, portal vein tumor thrombus. Bold italics indicate statistically significant values. *P<0.05
